# Characteristics and outcomes of vitrectomy for proliferative diabetic retinopathy in young versus senior patients

**DOI:** 10.1186/s12886-020-01688-3

**Published:** 2020-10-19

**Authors:** Mengyu Liao, Xiaohong Wang, Jinguo Yu, Xiangda Meng, Yuanyuan Liu, Xue Dong, Jianan Li, Rodrigo Brant, Bo Huang, Hua Yan

**Affiliations:** 1 Department of Ophthalmology, Tianjin Medical University General Hospital, No.154 Anshan Road, Tianjin, 300052 China; 2 Laboratory of Molecular Ophthalmology, Tianjin Medical University, Tianjin, China; 3 Department of Ophthalmology and Visual Sciences, Federal University of São Paulo, São Paulo, Brazil; 4 Department of Ophthalmology, University of Mississippi Medical Center, Jackson, USA

**Keywords:** Proliferative diabetic retinopathy, Vitrectomy, Young patients, Neovascular glaucoma

## Abstract

**Background:**

Proliferative diabetic retinopathy (PDR) is one of the most common cause of vision loss in diabetic patients, and the incidence age of PDR patients gradually gets younger. This study aims to compare the characteristics of PDR and outcomes following vitrectomy in young and senior patients.

**Methods:**

This is a retrospective case series study. Data of 116 eyes of 92 patients who underwent vitrectomy for PDR from February 2012 to February 2017 were reviewed, which were divided into young and senior patient groups. All patients were followed up for 24 months at least.

**Results:**

There were 62.1% of eyes with tractional retinal detachment secondary to PDR in the young patient group, while only 12.1% of eyes in the senior patient group with this surgery indication. (*P* < 0.001) The best corrected visual acuity increased in 41 eyes (70.7%), stable in 9 eyes (15.5%), and decreased in 8 eyes (13.8%) in young patients at the final follow-up. And it increased in 47 eyes (81.0%), stable in 2 eyes (3.4%), and decreased in 9 eyes (15.5%) in senior patients.(*P* = 0.085) Postoperative complications mainly included recurrent vitreous hemorrhage (24.1%), retinal detachment (3.4%), neovascular glaucoma (NVG) (27.6%) and nuclear sclerosis (53.4%) in young patients, and it was 19.0, 0.0, 1.7 and 3.4% in senior patients respectively.

**Conclusion:**

PDR of young patients is more severe than that of senior patients, and vitrectomy is an effective and safe method for PDR treatment. NVG is a main and severe complication besides nuclear sclerosis in young patients, and the incidence of NVG is higher compared to that in senior patients.

## Background

As the incidence of diabetes mellitus (DM) persistently rises in China, proliferative diabetic retinopathy (PDR) has become the most common cause of vision loss in diabetic patients [1, 2]. It was reported that the prevalence of diabetic retinopathy (DR) and sight-threatening DR was 27.9 and 12.6% in diabetic patient population in China [3]. Without timely treatment, the patients with PDR will suffer from significant decrease in vision and even blindness [4]. Although pars plana vitrectomy (PPV) is considered as an effective method in treating PDR, there are some adverse complications associated with the procedure, these include retinal detachment (RD), neovascular glaucoma (NVG), and recurrent vitreous hemorrhage (VH) [5].

Prolonged hyperglycemia in diabetic patients leads to retinal ischemia, which results in the formation and contraction of fibrovascular membrane (FVM) to cause tractional retinal detachment (TRD) [6]. *Arevalo* et al. showed that 5.2% patients with severe PDR developed or had progression of TRD [7]. While one previous study reported rhegmatogenous retinal detachment (RRD) occurred in 4.3% patients after PPV with or without tamponade for recurrent VH caused by PDR [8]. NVG is a common ocular complication secondary to PDR with neovascularization of the iris and anterior chamber angle. *Kwon* et al. reported in a recent study that the incidence of NVG after vitrectomy was 11.8% in patients with VH associated with PDR [9]. A retrospective review from a tertiary center in China showed that NVG comprised 5.8% of glaucoma patients, and DR was the commonest cause [10]. Postoperative recurrent VH following PPV for PDR is a common event, and delays visual recovery and can necessitate additional surgical management. Our previous study analyzed reasons for 10.2% (32/315 PDR eyes) with post-vitrectomy VH and found out the main reason was fibrovascular ingrowth [11]. *Sato* et al. reported incidences of early and late post-vitrectomy VH were 18.9 and 17.9%, respectively [12]. And the occurrence of early recurrent VH in another study was 24.3% without injection of ranibizumab [13].

The age at onset of diabetes is considered as a key factor in the development and progression of PDR. It was noted that young patients with PDR were at higher risk of visual loss than older patient population [14]. It has been shown in a recent study that young patients who received PPV for PDR presented with worse anatomical features at the time of surgery and had a higher risk of developing postoperative recurrent RD [15]. *Wong* et al. have postulated that the onset age of type 2 DM under 45 was an independent risk factor in the development of PDR after matching on the duration of diabetes and adjusting for traditional risk factors like metabolic control [16].

Through the retrospective analyses of clinical data in this study, we intend to find out the characteristics of PDR at different ages, and to evaluate surgical outcomes and visual prognosis following vitrectomy for PDR treatment in young patients as compared with senior patients.

## Methods

### Patients

Records of 116 eyes of 92 consecutive patients with a diagnosis of type 2 diabetes complicated PDR from February 2012 to February 2017, who have undergone vitrectomy in the ophthalmology department of Tianjin Medical University General Hospital were reviewed retrospectively. According to the definition of “young people” from World Health Organization, we designed the young patient group within an age range from 18 to 44 years old, and patients older than 45 years old as the senior patient group. This study was in accordance with the tenets of the Helsinki Declaration and was approved by Tianjin Medical University General Hospital Ethics Committee.

### Data collection

Preoperative records included the age at operation, gender, diabetic conditions, and systemic diseases (hypertension, cardiovascular disorders, cerebrovascular disorders and kidney disorders with dialysis). The blood glucose and blood pressure measured on the day of operation were recorded. Histories of previous panretinal photocoagulation (PRP) and preoperative intravitreal injection (IVI) of ranibizumab (Lucentis; Novartis Pharma Schweiz AG, Risch, Switzerland) in eyes with active neovascular membrane were also collected. The following data were mainly obtained from medical documents, including surgical procedures, intraoperative findings, anatomical outcomes, postoperative complications, best corrected visual acuity (BCVA) and intraocular pressure (IOP) at 1, 2, 6, 12, and 24 months postoperatively. All patients were followed up for 24 months at least.

Preoperative and postoperative ocular examinations included BCVA, IOP, slit-lamp examination, ocular fundus examination and ocular B-scan. Visual acuity (VA) was checked by the two-way visual chart both for decimal and LogMAR measurement. All patients had been examined in detail with dilated pupils. According to the Diabetic Retinopathy Preferred Practice Pattern Guidelines issued by American Academy of Ophthalmology, all patients were graded ‘High-risk PDR’. Exclusion criteria included eyes with no light perception before surgery, patients with severe systemic diseases, abnormal coagulant function, incomplete records of clinical and accessory examinations, eyes with previous intraocular surgery history, and eyes complicated with ocular trauma, ocular tumors, uveitis or other severe ocular diseases.

### Surgical procedure

All surgeries were performed by one surgeon under retrobulbar anesthesia using a standard three-port 23G vitrectomy. We used the vitreous cutter (Stellaris PC; Bausch & Lomb, Rochester, NY, USA). Phacoemulsification was performed in patients with cataract at the beginning of vitrectomy. During PPV, VH was removed as much as possible, and then triamcinolone acetonide (TA) was applied for offering a better identification to completely eliminating vitreous cortex and proliferative membranes. Remarkably, we had observed varying degrees of posterior vitreous detachment (PVD) in most of the eyes, including partial PVD and complete PVD, which was more severe in the senior group. Retinotomy was performed in cases with the extensive subretinal proliferation. Endolaser photocoagulation were done after air-fluid exchange, and silicone oil (Oxane 5700; Bausch & Lomb, Rochester, NY, USA) or C3F8 was used in cases with RD. Surgical records including intraoperative findings and postoperative complications were outlined in the Table 1.

**Table 1:** Clinical characteristics in young and senior patient groups

Characteristics	Young patient group	Senior patient group	*P*-value
Total eyes (patients)	58 (44)	58 (48)	0.359
Age at operation (years)	37.52 ± 5.80	57.60 ± 9.06	< 0.001
Gender (n, %)			
Male	28 (48.3%)	29 (50.0%)	0.853
Female	30 (51.7%)	29 (50.0%)	0.853
Diabetes mellitus			
History (years)	9.17 ± 6.49	13.98 ± 6.89	< 0.001
Therapy (n, %)			
Drugs only	10 (17.2%)	11 (19.0%)	0.809
Drugs and insulin	10 (17.2%)	22 (37.9%)	0.013
Insulin only	34 (58.6%)	16 (27.6%)	0.001
Unknown	4 (6.9%)	9 (15.5%)	0.141
Blood glucose (mmol/L)	6.79 ± 2.26	6.84 ± 2.11	0.905
Blood pressure (mmHg)			
Systolic blood pressure (mmHg)	149.28 ± 20.37	154.21 ± 22.27	0.197
Diastolic blood pressure (mmHg)	89.26 ± 9.77	81.22 ± 10.95	< 0.001
Systemic diseases			
Hypertension (n, %)	30 (51.7%)	25 (43.1%)	0.353
Cardiovascular disorders (n, %)	4 (6.9%)	9 (15.5%)	0.141
Cerebrovascular disorders (n, %)	6 (10.3%)	9 (15.5%)	0.406
Kidney disorders (n, %)	27 (46.6%)	12 (20.7%)	0.003
Renal dialysis (n, %)	6 (13.6%)	2 (4.2%)	0.692
Eyes (n, %)			
Right	31 (53.4%)	27 (46.6%)	0.458
Left	27 (46.6%)	31 (53.4%)	0.458
Preoperative BCVA (LogMAR)	1.79 ± 0.55	1.73 ± 0.56	0.536
Preoperative IOP (mmHg)	15.93 ± 6.16	14.83 ± 3.27	0.230
Preoperative PRP (n, %)	2 (3.5%)	3 (5.2%)	1.000
Preoperative IVI (n, %)	5 (8.6%)	12 (20.7%)	0.066
Surgical records			
Vitreous tamponade (n, %)			
C3F8	5 (8.6%)	6 (10.3%)	0.751
Silicone oil	36 (62.1%)	23 (39.7%)	0.016
BSS	17 (29.3%)	29 (50.0%)	0.023
Cataract extraction (n, %)	16 (27.6%)	13 (22.4%)	0.520
Retinotomy (n, %)	24 (41.4%)	10 (17.2%)	0.004
Intraoperative findings (n, %)			
VH only	0 (0.0%)	38 (65.5%)	< 0.001
FVM with or without VH	22 (37.9%)	13 (22.4%)	0.069
TRD with or without VH	36 (62.1%)	7 (12.1%)	< 0.001
Complicated with RVO	24 (41.4%)	20 (34.5%)	0.444
Complicated with optic atrophy	2 (3.4%)	0 (0.0%)	0.476
Early-postoperative inflammation	6 (10.3%)	1 (1.7%)	0.119
Postoperative complications (n, %)			
Recurrent VH	14 (24.1%)	11 (19.0%)	0.498
Recurrent RD	2 (3.4%)	0 (0.0%)	0.476
NVG	16 (27.6%)	1 (1.7%)	< 0.001
Nuclear sclerosis	31 (53.4%)	2 (3.4%)	< 0.001
Secondary surgery (n, %)	6 (10.3%)	3 (5.2%)	0.298

Values are presented as mean ± standard deviation

*BCVA* Best corrected visual acuity, *IOP* Intraocular pressure, *PRP* Panretinal photocoagulation, *IVI* Intraocular injection, *BSS* Balanced salt solution, *PPV* Pars plana vitrectomy, *VH* Vitreous hemorrhage, *FVM* Fibrovascular membrane, *TRD* Tractional retinal detachment, *RVO* Retinal vein occlusion, *RD* Retinal detachment, *NVG* Neovascular glaucoma

### Statistical analysis

Statistical analysis was performed by SPSS version 19.0 software, including t-tests, chi-square tests, and non-parametric tests. *P* < 0.05 was considered to have statistical significance. Data was presented as mean ± standard deviation or the percentages.

## Results

There were 58 eyes of 44 patients in young group with the average age of 37.5 years old, and 58 eyes of 48 patients in senior group with the average age of 57.6 years old. The mean duration of DM was 9.17 ± 6.49 years in young patients while 13.98 ± 6.89 years in senior patients when they were operated. (*P* < 0.001) Details of the clinical characteristics of all patients were listed in Table 1. When comparing their systemic diseases, the young group showed higher incidence of kidney disorders and tended to have higher diastolic blood pressure than senior group. Among the patients with kidney disorders, young patients had a higher incidence of renal dialysis (6 patients, 13.6%) than senior patients (2 patients, 4.2%), although without significant difference (*P* = 0.692). Although there was no remarkable difference in the occurrence of bilateral PDR between two groups, more young patients were with bilateral PDR.

As we can see from the surgical records, most of the young patients underwent vitrectomy mainly for the surgical indication of TRD, however, VH was the main reason for receiving PPV in senior patients. Retinal vein occlusion (RVO) was found more frequently in young patients with 24 (41.4%) eyes, and with 20 (34.5%) eyes in the senior patients, but there was no significant difference. (*P* = 0.444) None of the patients in senior patient group had optic atrophy, but 2 (3.4%) eyes in young patient group was noted with optic atrophy. (*P* = 0.476) Silicone oil tamponade (62.1%) accounted for the majority in young patients intraoperatively, in contrast, 39.7% senior patients received silicone oil tamponade. (*P* = 0.016) The distribution of C3F8 tamponade was similar between two groups. (*P* = 0.751) Retinotomy was performed in 24 (41.4%) eyes in young patient group and in 10 (17.2%) eyes in senior group. (*P* = 0.004) Cataract extraction was performed in 27.6 and 22.4% of young and senior patients respectively. (*P* = 0.520).

**Fig. 1:**
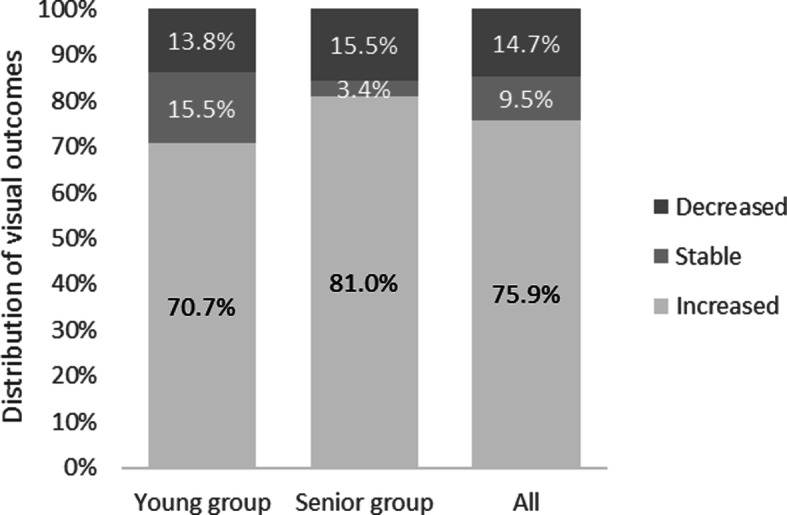
Distribution of visual outcomes between young and senior groups. Values were expressed as percentages. (*P* = 0.193)

**Fig. 2:**
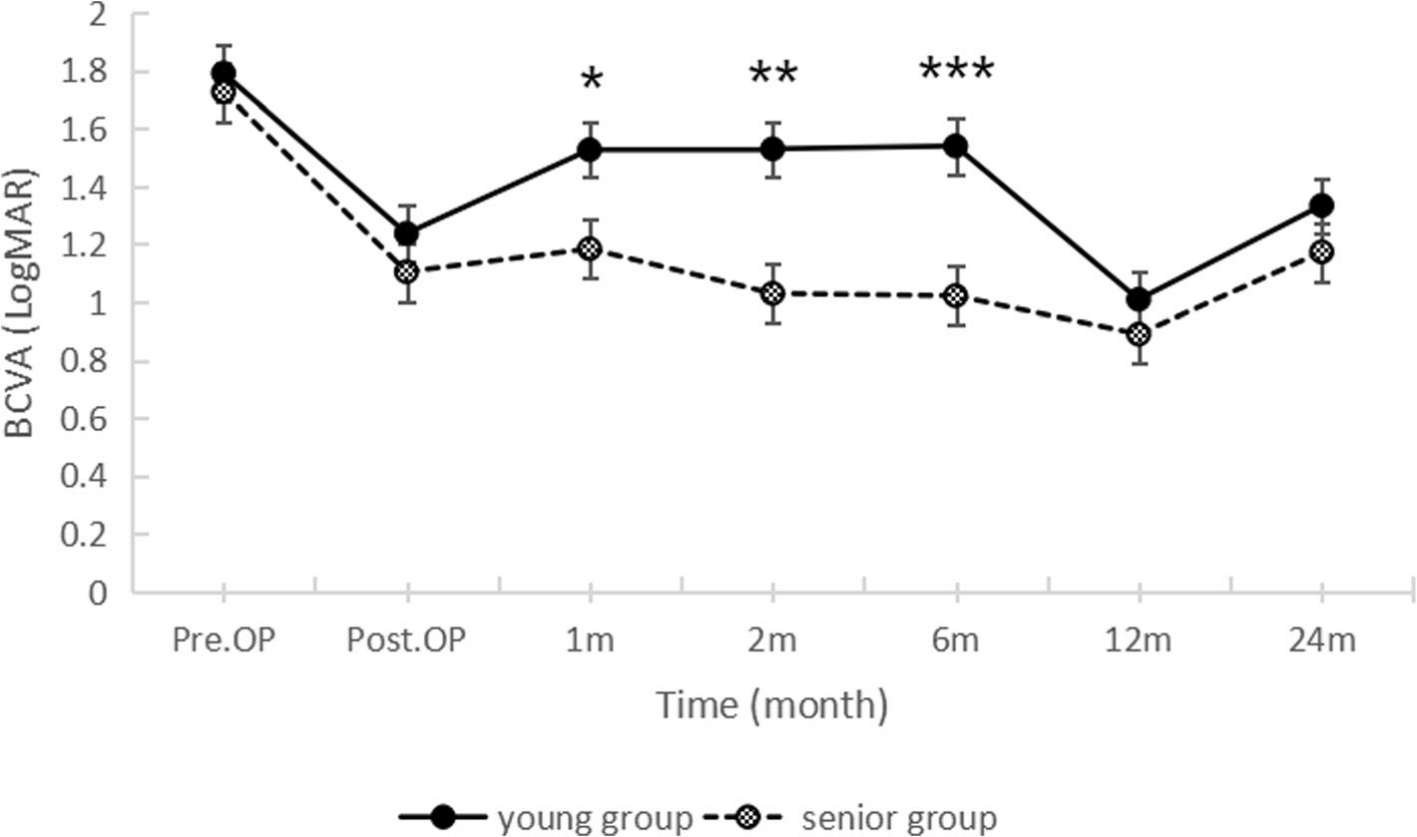
Changes of BCVA in young and senior patient groups at different time. Values are presented as mean ± standard deviation. (*P = 0.030; **P = 0.002 ***P = 0.003) BCVA = best corrected visual acuity, Pre.OP = preoperative visit, Post.OP = postoperative visit

**Fig. 3:**
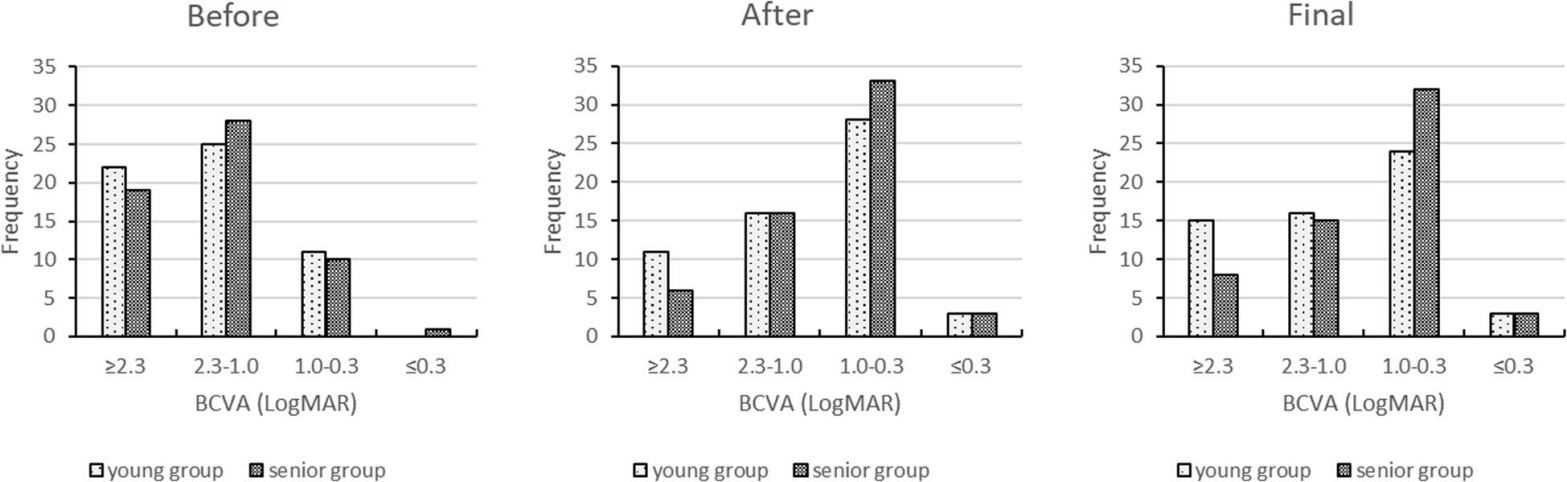
Distributions of BCVA in the eyes before, after the surgery, and at final follow-up time. Values are presented as frequency. BCVA = best corrected visual acuity

There were no significant differences in preoperative BCVA and IOP between two groups. (*P* = 0.536 and 0.230, respectively) The final BCVA increased in 41 eyes, stable in 9 eyes and decreased in 8 eyes in young patient group. In senior patient group, BCVA increased in 47 eyes, stable in 2 eyes and decreased in 9 eyes. As shown in Fig. 1, there was no significant difference in increased BCVA (*P* = 0.193) between two groups. The trends of BCVA changes at different time in two groups were shown in Fig. 2. And there was no significant difference in final BCVA between two groups (*P* = 0.085). As we can see from Table 2, the IOP was higher only after the surgery in young patient group. (*P* = 0.042) Distributions of BCVA in Fig. 3 showed that young patients had more eyes with low vision (≥2.3) whatever before, after the surgery, or at final follow-up.

**Table 2:** Changes of IOP in young and senior patient groups

	IOP (mmHg)		
	Before the surgery	After the surgery	Final follow-up
Young patient group	15.93 ± 6.16	17.59 ± 4.50	16.80 ± 5.45
Senior patient group	14.83 ± 3.27	16.10 ± 3.16	15.46 ± 9.15
*P*-value	0.230	0.042	0.340

Values are presented as mean ± standard deviation

*IOP* Intraocular pressure

Postoperative complications mainly included inflammatory response in anterior chamber, recurrent VH, RD, NVG, and nuclear sclerosis. Obvious inflammatory response in anterior chamber were found in 6 (10.3%) eyes in young patient group, and only 1 (1.7%) eye in senior patient group 1 week after the surgery. (*P* = 0.119) Sixteen (27.6%) eyes of the young patients developed NVG during follow-up period, and only one eye with NVG in senior patients. (*P* < 0.001) More patients were with nuclear sclerosis in young group (53.4%) than that in senior group (3.4%). (*P* < 0.001) The postoperative recurrent VH and RD showed no statistically significant difference between two groups. (*P* = 0.498 and 0.476, respectively) Overall, 6 eyes of young patient group required the secondary surgeries for the cause of recurrent VH with or without RD, and 3 eyes underwent secondary procedures for recurrent VH in senior patient group. (*P* = 0.298).

## Discussion

Previous studies have shown that the age of onset and the DM duration played an important role in the development and progression of DR [17]. We designed 45 years age as the cutoff point, consistent with the previous study which reported that the prevalence and severity of DR was significantly higher in the young-onset patients especially younger than 45 years old [16]. Young patient group had surgeries at earlier age (38 years) and shorter history of DM (9 years) in our study. There were more patients with severe proliferative lesions (FVM and TRD) in young group, and the preoperative BCVA of these patients was poor. Additionally, the systemic diseases of young patient group were worse than that of senior patient group, as can be seen in patients who had hypertension (51.7% vs 43.1%) and kidney disorders (46.6% vs 20.7%).

The effects of systemic risk factors on the development and severity of PDR in young patients have been noted. In young patient group, there was a higher rate of kidney disorders than senior patient group (*P* = 0.003). Despite of good diabetic control, DM can result in a variety of chronic complications due to the microvascular complications for human health, including both DR and nephropathy. The changes of microenvironment in retina is similar to glomerular microvessels, and diabetic kidney disorders in young patients often predict a rapid deterioration of the microvascular environment [18, 19]. The effects of hypertensive damage in PDR eyes were unknown, which was assumed that high blood pressure damaged the retinal capillary endothelial cells [20]. *Kostraba* et al. reported a group of PDR patients aged 18–29 years had higher diastolic blood pressure, which was in agreement with the results of young patients in our study [21].

In this study, one of the most important surgical technique was the application of TA intraoperatively. TA is a kind of synthetic corticosteroid with the function of accumulating platelet and fibrinogen, which was usually applied to PPV for clear identification of vitreous and epiretinal membrane. In our study, TA was injected into vitreous cavity just after most of the vitreous removed that made it easily stain the remaining vitreous and epiretinal membrane for completely removing the epiretinal membrane. Meanwhile, in case of the bleeding induced by epiretinal membrane peeling, TA particles could cover the bleeding area and form a thin membrane with red blood cells to manage the bleeding and provide a clear view [22].

It was confirmed that vitreous collagen fibrils changed with aging, these changes could include vitreous liquefaction and weakening of vitreoretinal adhesion, which combined with PVD [23]. The classic theory was acute vitreoretinal separation firstly at the posterior pole, and then peripherally [24]. *Gella* et al [25] reported that risk factors for PVD included age and sight-threatening DR. In PDR patients, the new vessels are pulled forward by the contracting vitreous gel and grow along the posterior surface of the detached vitreous cortex. As a result, VH was more often in the senior patients. However, there were peripheral firmly vitreoretinal adhesion and actively extensive proliferation in young PDR patients. Therefore, complete removal of them seemed to be particularly important to avoid TRD. Laser photocoagulation was performed to seal retinal holes intraoperatively to avoid the over proliferation of retina induced by cryotherapy. With the indications of active neovascularization in retina and/or iris, injection of ranibizumab 3–5 days preoperatively was found to help reduce bleeding during the surgery, because it can cause a short duration of the regression of neovascularization secondary to PDR [26]. We found that retinotomy was performed more in young patients, and the possible reason was more subretinal proliferative membrane. Laser photocoagulation has been proved to be an effective therapy for DR and PDR, so panretinal endolaser photocoagulation was performed for almost every patient in this study.

In our study, young patients were noted to have a higher rate of complications after the vitrectomy. The incidence of postoperative NVG was 27.6 and 1.7% in two groups in our study respectively. The possible reasons are that young patients have the tendency to have IOP spikes after the surgery that cause ischemia to the retina. One study concluded that elevated IOP and usage of retinal tamponade during retinal surgery were identified as the risk factors of NVG, which supported the evidences in our study [27]. NVG developed as a result of ischemic ocular pathologies like PDR. Early study had examined intense anti-proliferative surgery as a treatment for advanced NVG [28, 29]. As a result, it was essential to perform sufficient anti-proliferative PRP during the vitrectomy, especially in young patients. In addition to NVG, postoperative nuclear sclerosis was found to be the most common complication after vitrectomy in young patients (53.4%) during 2-year follow-up period, in comparison, an overall 35% of the patients had cataract surgery in 2 years in *Steel DH’s* study [30]. Previous studies have shown that cataract was more common in diabetic patients, and the commonest type was nuclear sclerosis [31].

The major limitation of our study was relatively small sample size. In addition, we were unable to track some of the associated clinical data, such as the levels of HbA1c and blood lipids, body mass index (BMI), long-term metabolic control. and some other ocular conditions such as phakic status and the exact time of silicone oil removal, etc. Lack of quantification of PVD status was a limitation to compare the age-related differences. Finally, the retrospective nature of the study did not allow for a more accurate comparison between the two groups under controlled conditions. However, because of the limited previously published reports in this field, our study provided important observations and insights on the characteristics of PDR and outcomes following vitrectomy for PDR in young versus senior patients.

## Conclusions

In summary, vitrectomy is an effective method for treatment of severe PDR in young patients. However, the outcomes are limited and worse than that in treating senior patients. Our data showed PDR of young patients was more severe than that of senior patients, whatever in baseline or post-operation, NVG was a main and severe complication besides nuclear sclerosis in young patients, and the incidence of NVG was higher compared to that in senior patients.

## Data Availability

The datasets used and/or analysed during the current study available from the corresponding author on reasonable request.

## Abbreviations

PDR: Proliferative diabetic retinopathy
DM: Diabetes mellitus
DR: Diabetic retinopathy
PPV: Pars plana vitrectomy
RD: Retinal detachment
NVG: Neovascular glaucoma
VH: Vitreous hemorrhage
FVM: Fibrovascular membrane
TRD: Tractional retinal detachment
PRP: Panretinal photocoagulation
IVI: Intraocular injection
BCVA: Best-corrected visual acuity
IOP: Intraocular pressure
VA: Visual acuity
TA: Triamcinolone acetonide
PVD: Posterior vitreous detachment
RVO: Retinal vein occlusion
BMI: Body mass index
